# 

**Published:** 1889-09-14

**Authors:** 


SATURDAY, SEPTEMBER 14th, 1889.

**The Medical Freshman** - 367
**Within the Wards.**—The Diagnosis of Cancer - 368
**A Dean's Opinion.**—An Interview with Mr. A. Pearce Gould 369
**The Student as the Nurse sees him** - 370
**The Doctor's Pulpit.**—The Medical Mind and Conscience 371
**"Mems." for Muddled Medicals** - 372
**Getting into Practice** - 373
**Notes and News** - 374
**Everybody's Page** - 376
**Annotations.**—Coaxing Sleep—Toughened Nerves—Worry, Work, and Sunstroke - 377
**The Medical Schools of London.**—Arrangements for the Session, 1889-90 - 378
**The Unqualified Assistant** - 380
**Her Heart's Mistake.** By E. D. Cumming. Chap. xxii. - 381
**Amusements and Relaxation** - 382

**EXTRA SUPPLEMENT—THE NURSING MIRROR.**

**En Passant.**—Lady Doctors—Doll Competition—Registration—Massage—Seamen's Hospital—Sick Children in Holland—Charity and Religion—Air Space - xci
**Physiology for Nurses.** No. 9.—Dislocations - xcii
**Bunchy** - xcii
**The Children's Hospital** - xciii
**Everybody's Opinion.**—Superannuated at Forty-Three - xciii
**Hymn for a Hospital** - xciv
**Vacancies** - xciv
**Notes and Queries** - xciv

